# Avoiding the Health Hazard of People from Construction Vehicles: A Strategy for Controlling the Vibration of a Wheel Loader

**DOI:** 10.3390/ijerph14030275

**Published:** 2017-03-08

**Authors:** Feng Chi, Jun Zhou, Qi Zhang, Yong Wang, Panling Huang

**Affiliations:** 1 School of Mechanical Engineering, Shandong University, Jinan 250061, China; feng.chi@sdlg.com.cn (F.C.); mewang@sdu.edu.cn (Y.W.); hfpl@sdu.edu.cn (P.H.); 2 Key Laboratory of High Efficiency and Clean Mechanical Manufacture, Ministry of Education, Shandong University, Jinan 250061, China; 3 Shandong Lingong Construction Machinery Co., Ltd., Linyi 276004, China; qi.zhang@sdlg.com.cn

**Keywords:** earthmoving machinery, vibration control, vibration transmission path, order tracking technique

## Abstract

The vibration control of a construction vehicle must be carried out in order to meet the aims of sustainable environmental development and to avoid the potential human health hazards. In this paper, based on market feedback, the driver seat vibration of a type of wheel loader in the left and right direction, is found to be significant over a certain speed range. In order to find abnormal vibration components, the order tracking technique (OTT) and transmission path analysis (TPA) were used to analyze the vibration sources of the wheel loader. Through this analysis, it can be seen that the abnormal vibration comes from the interaction between the tire tread and the road, and this is because the vibration was amplified by the cab mount, which was eventually transmitted to the cab seat. Finally, the seat vibration amplitudes were decreased by up to 50.8%, after implementing the vibration reduction strategy.

## 1. Introduction

With the rapid development of the construction machinery industry in China and the requirements for products of a higher quality, customers are paying more for such products and more attention is being paid to the comfort of the construction equipment. Vibration transfers to the seat and the human body are known to be a major source of discomfort for the driver. Therefore, these vibrations are an important issue to consider, having a significant influence on human fatigue and safety [1].

A vibration of any frequency which is transmitted to the body is called a Whole Body Vibration (WBV). WBV has been recognized as a health hazard to vehicle drivers [2,3,4,5] because it can lead to fatigue and injury. Construction vehicle drivers are often exposed to WBV for long periods of time, sometimes longer than 8 h a day, under severe construction site conditions [6]. The vibrations are transmitted to the body of the driver through excited parts of the vehicle, including the frame and cab seat [7]. Experimental studies have found that exposure to WBV can affect the lumbar spine and the connected nervous system [8], causing lower back pain [9]. Internal organ disorders caused by WBV can be significantly attributed to the resonance excitation of individual organs (the natural frequencies of most organs are between 2 and 18 Hz) [10]. WBV may also cause speech modulation, and can significantly affect body movement and the ability to complete the task, according to the British Standards Institution [11].

In addition, vibrations may cause early fatigue failure of some parts of construction machineries and vehicles, and shorten their service lives, resulting in a waste of energy and resources. Therefore, in order to improve the competitiveness, the manufacturers of construction machinery are, more than ever, aware of the importance of safety, comfort, and vibration and noise quality, in addition to the operational capacity. Vibration reduction has become the inevitable development trend of the construction machinery industry. The first step toward vibration reduction is to diagnose the vibration sources and transmission paths, which are referred to as the fault diagnosis.

Different methods for identifying vibration sources have been proposed in previous works. Conventionally, frequency spectrum analysis is used for signal analysis in fault diagnosis. However, this method is not always effective when applied to situations involving various operation speeds and non-stationary vibration signals [12]. Another analysis technique for fault diagnosis using non-stationary signals, such as wavelet analysis, has been proposed [13]. The order tracking technique (OTT) is also a method for diagnosing a fault in a rotating machine [14]. Essentially, the OTT is used to transform a non-stationary signal in the time domain, into a stationary signal in an angular domain, thereby highlighting the vibration information related to the rotation speed, while restraining unrelated information [15].

The purpose of the present study was to identify the vibration sources of a cab seat in a wheel loader through a combination of tests and theory analyses, and to develop strategies for controlling vibration.

## 2. Vibration Transmission of the Seat Vibration for Wheel Loader

**Figure 1:**
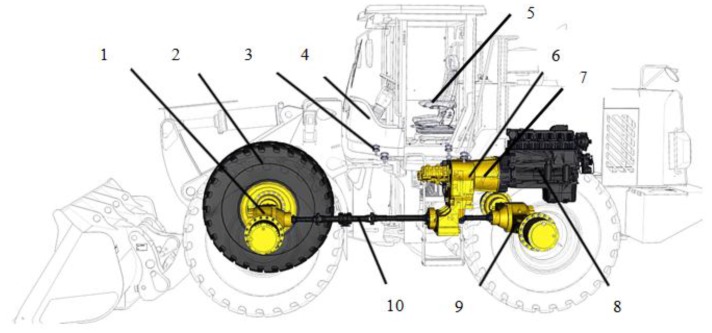
The power transmission path: 1. Front axle; 2. Tire; 3. Cab damper; 4. Cab; 5. Cab seat; 6. Transmission assembly; 7. Hydraulic torque converter; 8. Engine; 9. Rear axle; 10. Transmission shaft.

A wheel loader is an important type of earth-moving machinery that is mainly used to load materials in severe environments, such as mining and construction fields, etc. [16]. Its power transmission path is shown in Figure 1. The output torque of engine 8 is transmitted to the front axle 1 and the rear axle 9 through the hydraulic torque converter 7, the transmission assembly 6, and then through the transmission shaft 10. Finally, the power drives the wheel loader to work.

The vibration sources of the cab seat during walking are the engine vibration, tire/road vibration, and other vibrations associated with the machinery.

### 2.1. Engine Vibration

**Figure 2:**
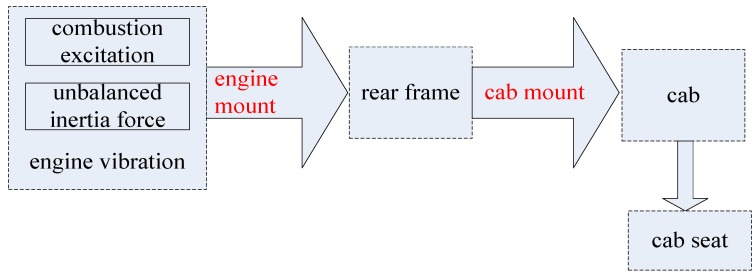
The vibration transmission path of an engine.

Engine vibration is caused by combustion excitation and the unbalanced inertia force of the engine. Its vibration is transmitted from the engine mount, rear frame, and cab mount, to the cab, finally causing the cab seat to vibrate. Its transmission path is shown in Figure 2.

### 2.2. Tire/Road Vibration

Tire/road vibration is a result of the interaction between the tire and the road. The vibration caused by road irregularity and the elastic deformation of the tire during rolling, is transmitted from the tire, the driving axle, and the frame, to the cab and cab seat. The contribution of each mechanism to the overall tire/road vibration varies, depending upon the tire type and size (including the tire tread number and tread shape), tire pressure, the road or pavement, and the vehicle’s operating condition (including driving speed and load etc.).

### 2.3. Other Vibration

In addition to the above two sources, the vibration of the main relief valve, hydraulic pump, hydraulic pipeline, and transmission parts, can all be transmitted to the cab seat via the frame and cab.

## 3. Cab Seat Vibration Test and Order Analysis

Based on market feedback, the cab seat vibration of the wheel loader in the lateral direction (*y*-axis), is significant when the loader is driven on a hard road at speeds of 7.1–8.4 km/h, with a heavy load. Therefore, a vibration test will be conducted in order to further analyze the vibration source and reduce the vibration of the cab seat.

### 3.1. Cab Seat Vibration Test

#### 3.1.1. Test Condition

A vibration test was thus conducted on the wheel loader, while carrying a 5-ton load on a hard cement road. The driving speed of the loader was slowly increased from idle to forward speed in 1st gear. The pressure of the front tires was 0.45 MPa and that of the rear tires was 0.32 MPa.

#### 3.1.2. Sensors Arrangement and Data Collection

**Figure 3:**
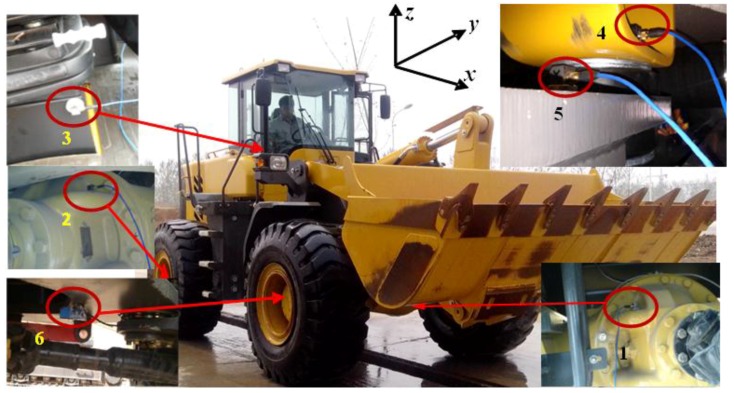
Vibration test site: 1—Front axle housing, 2—Rear axle housing, 3—Seat bracket, 4—Before cab mount, 5—After cab mount, 6—Front axle input shaft.

The vibration sources were identified by considering the relationship between the cab seat vibration and the rotating member excitation frequency employed in the test. Therefore, the three direction accelerometers are fixed in each key component, in order to measure the vibration acceleration at each measuring point. Figure 3 shows the test site. Five accelerometers (labeled 1–5, type 356A12) (PCB Piezotronics Inc., New York, NY, USA) were fixed to different components of the transmission system. A photoelectric sensor (labeled 6, type WL18-3P130F29) (Hydrotechnik GmbH, Limburg, Germany) was used to determine the rotational speed of the front-axle input shaft, and this was used as the tracking signal. In the figure, the *x*-, *y*-, and *z*-axis, represent the fore-and-aft, lateral direction, and vertical direction of the wheel loader, respectively. The data collection device (type Scada-05) and data analysis system (type CADA-X) were from LMS Company in Belgium.

### 3.2. Order Tracking Analysis

Table 1 gives the rotational frequencies and orders of the principal excitation sources, where *n* is the rotational speed of the front-axle input shaft, determined by the photoelectric sensor. This rotational speed was assumed to be of an order of 1. The reduction gear ratio between the driving-axle output shaft and the front-axle input shaft was 22.85, and the number of tire treads was 28.

### 3.3. Results and Discussion

#### 3.3.1. Test Results

**Figure 4:**
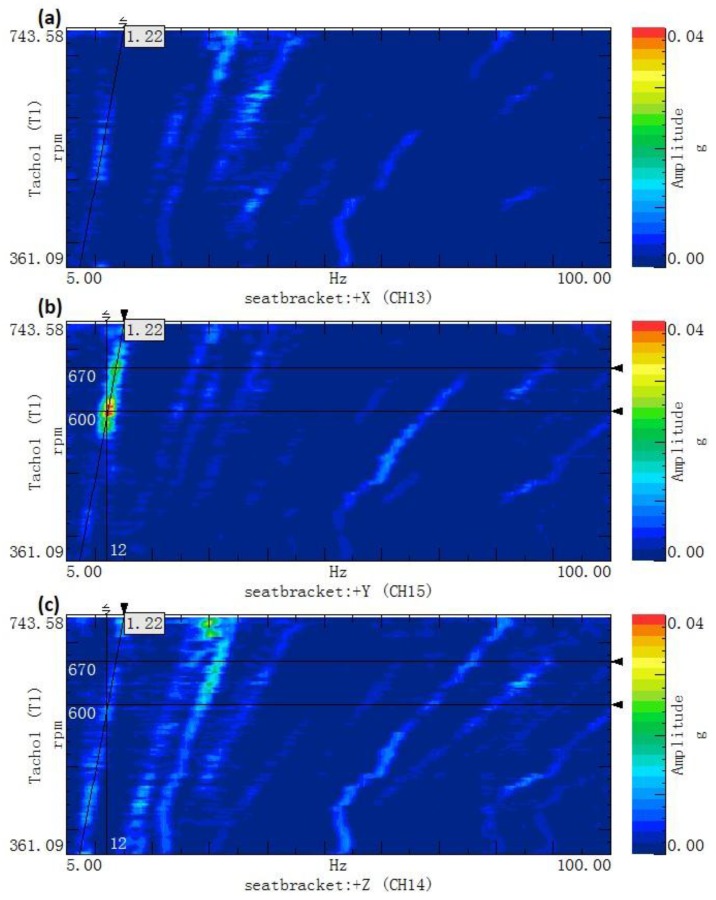
The order tracking colormap of the acceleration: (**a**) *x* direction; (**b**) *y* direction and (**c**) *z* direction.

Figure 4 shows the order tracking colormap of the acceleration at the driver’s seat in the three directions. The axs respectively represent the vibration frequency (unit: Hz) and the speed of the front-axle input shaft (unit: rpm). The distribution of the vibration energy is reflected in the colormap by a scale of different colors.

From the figure, in the 1.22 order, the vibration energy is more concentrated when the rotational speed is between 580 and 630 rpm, which corresponds to driving speeds between 7.1 and 8.4 km/h. This trend is most evident in the *y*-direction, when studying the colormap. According to Table 1, the source of the cab seat vibration is the tire thread, because it corresponds to the 1.225 order.

#### 3.3.2. Transmission Path Analyses

**Figure 5:**
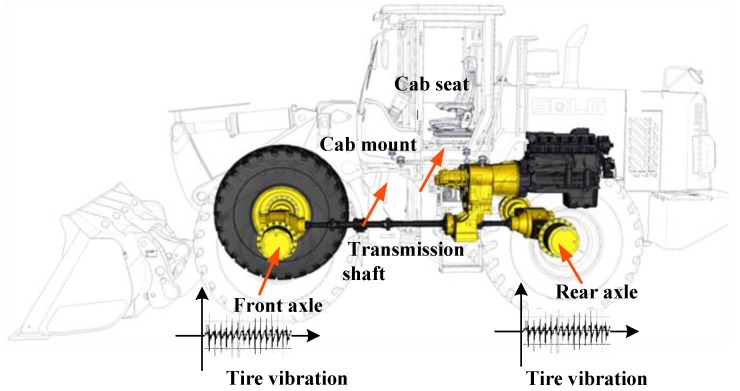
Transmission path analysis.

Figure 5 shows the TPA between the tires and the cab seat. The tire vibrations are transmitted to the cab seat through the front and rear axles, transmission shaft, vehicle frame, and cab mount.

**Figure 6:**
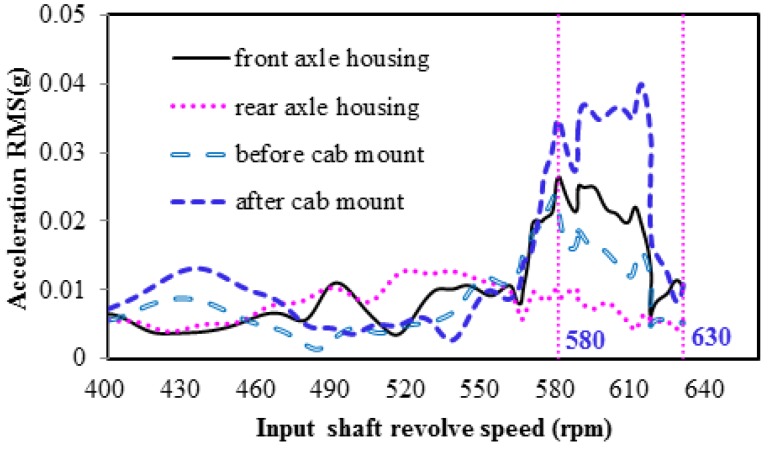
Comparison of 1.22 order vibration in the *y*-direction.

Figure 6 shows the 1.22 order vibration acceleration of the four transmission parts, with different rotational speeds in the *y*-direction. As can be seen in the figure, the results show a rotational speed (*n*) of between 580 and 630 rpm (approximately corresponding to tire vibration frequencies of between 11 and 13 Hz, as determined by Equation (1)). The vibration amplitude of the front axle is far higher than that of the rear axle, because it bears load in front of the loader. The vibration amplitude in front of the cab mount is equivalent to that of the front axle. However, it is obvious that the vibration amplitude behind the cab mount is considerable, indicating that the cab mount has an amplification effect on the vibration in the *y*-direction. Therefore, there is a need for further study, to optimize the structure of the cab mount.

(1) $$f=\frac{n}{22.85\times 60}\times 28$$

#### 3.3.3. Vibration Reduction Theories

**Figure 7:**
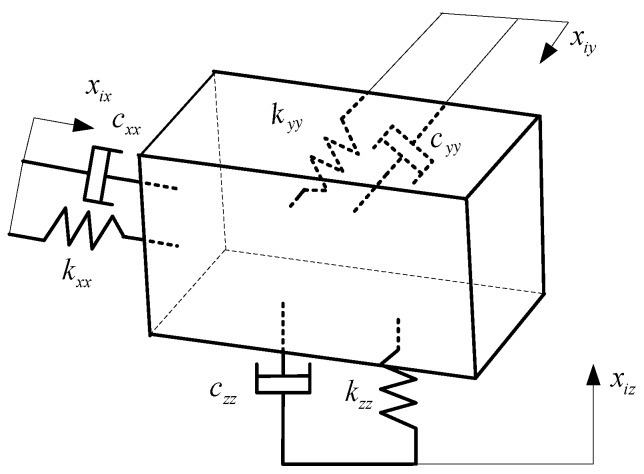
Vibration model of the cab mount system.

Let us assume that the vibration model of the cab mount is a dynamic system, as shown in Figure 7. The motion equations of the system were formulated as follows, based on Newton’s second law:

(2) $$\left[M]{\ddot{x}}_{o}(t)+[C]{\dot{x}}_{o}(t)+[K]x_{o}(t)=[C]{\dot{x}}_{i}(t)+[K]x_{i}(t\right)$$

where *[M]*, *[C]*, and *[K]* matrices are given as:

$$\begin{array}{ccc} M=\left|\begin{array}{c} \begin{array}{ccc} m_{x} & 0 & 0\\ 0 & m_{y} & 0\\ 0_{x} & 0 & m_{z} \end{array} \end{array}\right| & C=\left|\begin{array}{ccc} c_{xx} & c_{xy} & c_{xz}\\ c_{yx} & c_{yy} & c_{yz}\\ c_{zx} & c_{zy} & c_{zz} \end{array}\right| & K=|\begin{array}{ccc} k_{xx} & k_{xy} & k_{xz}\\ k_{yx} & k_{yy} & k_{yz}\\ k_{zx} & k_{zy} & k_{zz} \end{array}|\\ x_{o}=\left|\begin{array}{c} x_{ox}\\ x_{oy}\\ x_{oz} \end{array}\right| & x_{i}=\left|\begin{array}{c} x_{ix}\\ x_{iy}\\ x_{iz} \end{array}\right|=\left|\begin{array}{c} 0\\ 0\\ x_{iz} \end{array}\right| & \end{array}$$

where *x*_i_ (*t*) and *x*_o_ (*t*) are the vibration displacements in front of the cab mount (the input signal) and behind the cab mount (the output signal), respectively; [*M*] is the mass matrices of the system, including the cab and driver etc.; [*K*] is the stiffness matrices; and [*C*] is the damping coefficient matrices of the system.

Because the cab seat vibration in the lateral direction (*y*-axis) is significant, only the transfer function of the cab mount in the *y*-axis was analyzed, as follows:

Assume that the input displacement is in the *x*- and *y*-direction because of the transfer direction of the tire vibration. So, *x_ix_* = 0, *x_iy_* = 0. The transfer function in the *y*-direction was computed by Formula (3):

(3) $$G_{zy}(s)=\frac{X_{oy}(s)}{X_{iz}(s)}=\frac{c_{yz}s+k_{yz}}{m_{y}s^{2}+c_{yy}s+k_{yy}}$$

where *X_oy_* (*s*) and *X_iz_* (*s*) are the Laplace transforms of *x_oy_* (*t*) and *x_iz_* (*t*), respectively.

**Figure 8:**
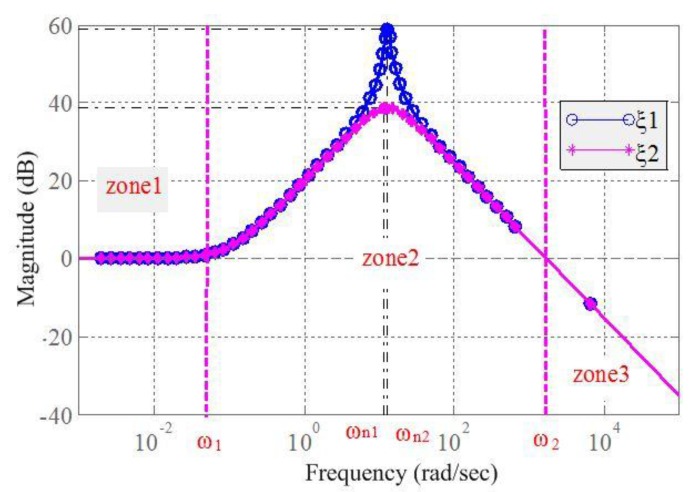
Bode diagram of the cab mount system (ξ2 < ξ1).

Figure 8 shows the Bode Diagram (amplitude) of the transfer function, where the *x*-axis represents the frequency and the *y*-axis represents the amplitude ratio between the input and output. In zone 1 (0–ω_1_), namely in low frequency, the ratio of the output to the input is approximately stable. In zone 2 (ω_1_–ω_2_), the output value was substantially amplified, and moreover, when the excitation frequencies are close to natural frequency ω_n_, the amplitude reaches a maximum value; namely, the resonance amplitude. In zone 3 (ω_2_–∞), namely in high frequency, the output value monotonically decreases. According to the phenomenon described in Section 3, the excitation frequency range of the tires is in zone 2, because the output amplitudes were significantly more enlarged than those of the input.

Using Equation (3), the damping ratio ξ and the undamped natural frequency ω_n_ can be calculated by the following equations:

(4) $$\xi =\frac{c_{yy}}{2}\sqrt{\frac{1}{m_{y}k_{yy}}},\;\omega_{n}=\sqrt{k_{yy}/m_{y}}$$

According to Figure 8, the maximum peak is around the natural frequency ω_n_, and the output can be magnified when the excitation frequencies are between ω_1_ and ω_2_. Furthermore, the greater the damping ratio ξ is, the smaller the peak value will be.

The vibration reduction method employed in the present study can thus be summarized as follows:

(1). Increase the system damping ratio ξ to reduce the peak output amplitude.

(2). Change the frequency ranges in zone 2 to avoid the excitation frequency (the excitation frequency of the tires in the present study was between 11 and 13 Hz).

The above analysis and Equation (4) indicate that the stiffness *k_yy_* of the cab mount should be decreased, to increase ξ and to decrease ω_n_. By this measure, the output amplitude can be reduced and the frequency ranges in zone 2 can be shifted to the left, which can cause the excitation frequency (it is considered to be no change) of the tires to shift to zone 3, because of the left shift of the whole bode curve, as shown in Figure 8; where the output amplitude is significantly reduced.

#### 3.3.4. Validation Tests

**Figure 9:**
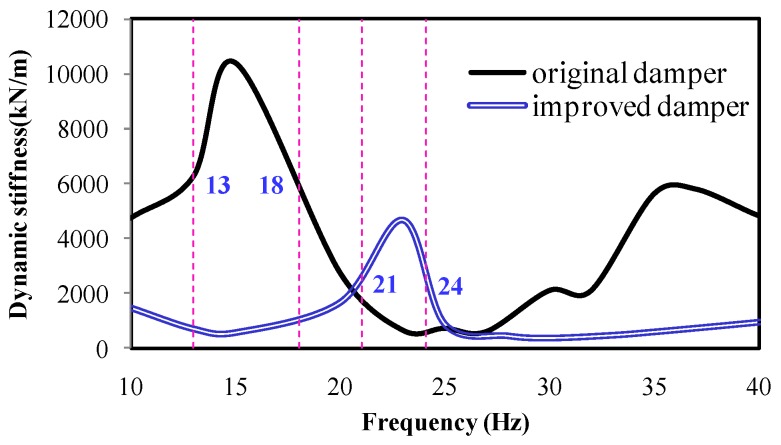
Dynamic stiffness comparison of the cab absorber.

In this paper, a method of replacing the cab absorber is adopted for reducing the stiffness *k_yy_* of the cab mount system. Figure 9 compares the original and improved stiffness of the cab absorber. It can be seen from the figure that the original cab mount exhibited a higher elastic coefficient, within 11–13 Hz.

**Figure 10:**
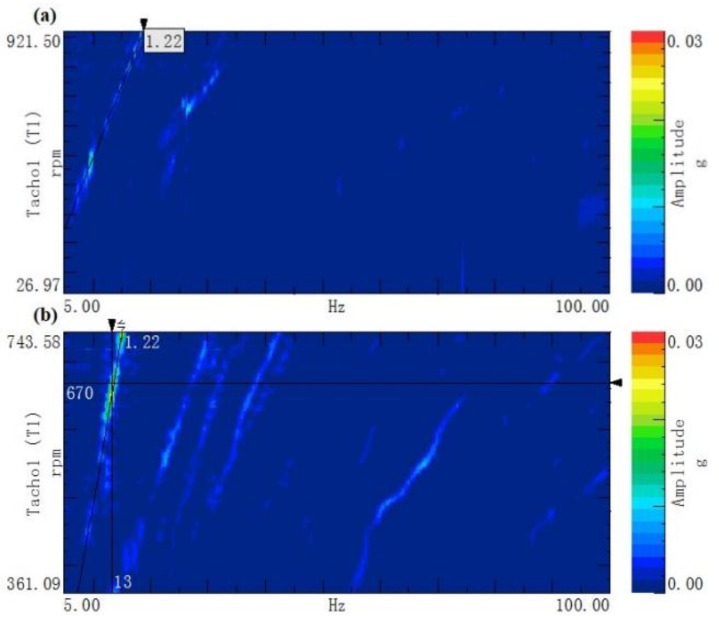
The order tracking colormap of the acceleration in *y*-direction: (**a**) improved cab mount and (**b**) original cab mount.

Figure 10 shows the spectral colormap comparison of the seat vibration with two cab dampers in the *y*-direction, under the same test conditions. From this figure, the 1.22 order vibration power of the cab seat is significantly decreased when using the improved cab mount. Additionally, the whole body vibration is weakened, according to the subjective feeling of operation under the same conditions.

**Figure 11:**
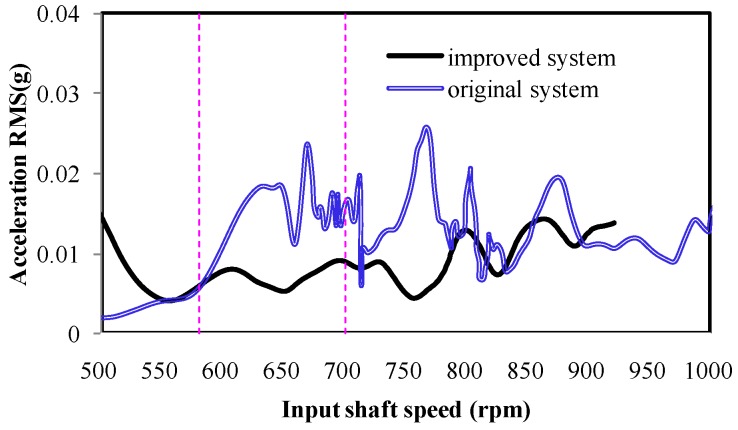
A vibration comparison of the cab seat at an order of 1.22.

Figure 11 shows the *y*-direction acceleration amplitudes of the cab seats of the two different cab mount systems (improved system and original system), at an order of 1.22. The improved cab mount obviously decreased the vibration amplitude during rotational speeds of 580–630 rpm. The statistical results indicate that the average vibration amplitude was decreased by 50.8% in this rotational speed range.

## 4. Conclusions

Methods for reducing the vibration of a wheel loader were investigated in this study, aimed at enhancing the market competitiveness of the machine and reducing the associated health risks. This is a summary of the study and the conclusions drawn from the findings:

(1) A frequency with an order of 1.22 was determined to be the main excitation frequency of the cab seat at rotational speeds of 580–630 rpm, and it originated from the interaction between the tires and the road.

(2) A vibration reduction mechanism was developed using a Bode Diagram of the cab mount system. The dynamic stiffness of the cab mount was reduced to decrease the output amplitude and shift the tire excitation frequency to the vibration reduction zone.

## Figures and Tables

**Table 1:** The rotational frequency and order of three excitation sources.

Excitation Source	Rotational Speed (rpm)	Frequency (Hz)	Order
Front axle input shaft	*n*	*n*/60	1
Driving axle output	*n*/22.85	*n*/22.85/60	1/22.85
Tire thread		*n*/22.85/60 × 28	1.225

## References

[1] Wu J.D., Chen R.J. (2004). Application of an active controller for reducing small-amplitude vertical vibration in a vehicle seat. J. Sound Vib..

[2] Langer T.H., Iversen T.K., Andersen N.K., Mouritsen O.Ø., Hansen M.R. (2012). Reducing whole-body vibration exposure in backhoe loaders by education of operators. Int. J. Ind. Ergon..

[3] Blood R.P., Rynell P.W., Johnson P.W. (2012). Whole-body vibration in heavy equipment operators of a front-end loader: Role of task exposure and tire configuration with and without traction chains. J. Saf. Res..

[4] Zhao X., Schindler C. (2014). Evaluation of whole-body vibration exposure experienced by operators of a compact wheel loader according to ISO 2631-1:1997 and ISO 2631-5:2004. Int. J. Ind. Ergon..

[5] Roseiro L.M., Neto M.A., Amaro A.M., Alcobia C.J., Paulino M.F. (2016). Hand-arm and whole-body vibrations induced in cross motorcycle and bicycle drivers. Int. J. Ind. Ergon..

[6] Kuijt-Eversa L.F.M., Krause F., Vink P. (2003). Aspects to improve cabin comfort of wheel loaders and excavators according to operators. Appl. Ergon..

[7] Cvetanovic B., Zlatkovic D. (2013). Evaluation of whole-body vibration risk in agricultural tractor drivers. Bulg. J. Agric. Sci..

[8] Bovenzi M., Hulshof C.T.J. (1998). An updated review of epidemiological studies on the relationship between exposure to whole-body vibration and low back pain. J. Sound Vib..

[9] Bovenzi M. (1996). Low back pain disorders and exposure to whole body vibration in the workplace. Semin. Perinatol..

[10] Koradecka D. (2010). Handbook of Occupational Safety and Health.

[11] British Standards Institution Guide to Measurement and Evaluation of Human Exposure to Whole-Body Mechanical Vibration and Repeated Shock. http://shop.bsigroup.com/ProductDetail/?pid=000000000000171912.

[12] Wu J.-D., Chen J.-C. (2006). Continuous wavelet transform technique for fault signal diagnosis of internal combustion engines. NDT E Int..

[13] Tse P.W., Yang W.X., Tam H.Y. (2004). Machine fault diagnosis through an effective exact wavelet analysis. J. Sound Vib..

[14] Bai M.R., Jeng J., Chen C. (2002). Adaptive order tracking technique using recursive least-square algorithm. J. Vib. Acoust..

[15] Cheng J.S., Zhang K., Yang Y. (2012). An order tracking technique for the gear fault diagnosis using local mean decomposition method. Mech. Mach. Theory.

[16] Zhou J., Huang P.L., Zhu Y.G., Deng J.X. (2012). A quality evaluation model of reuse parts and its management system development for end-of-life wheel loaders. J. Clean. Prod..

